# Clinical Profile and Diagnostic Spectrum of Autoimmune Comorbidities in Juvenile Idiopathic Arthritis: A Descriptive Single-Centre Observational Study

**DOI:** 10.3390/diagnostics16152381

**Published:** 2026-07-29

**Authors:** Alina Mariela Murgu, Adriana Mihai, Paula Popovici, Ninel Revenco, Mara Russu, Laura Mihaela Trandafir, Elena Țarcă, Dana-Teodora Anton-Păduraru, Alina Onofrei, Răzvan Popovici, Codrina Ancuța

**Affiliations:** 1 Grigore T. Popa University of Medicine and Pharmacy, 700115 Iași, Romania; alina.murgu@umfiasi.ro (A.M.M.); paula.popovici@umfiasi.ro (P.P.); mara.russu@umfiasi.ro (M.R.); laura.trandafir@umfiasi.ro (L.M.T.); tarca.elena@umfiasi.ro (E.Ț.); dana.anton@umfiasi.ro (D.-T.A.-P.); alina-alexandra.onofrei@email.umfiasi.ro (A.O.); razvan.popovici@umfiasi.ro (R.P.); codrina.ancuta@umfiasi.ro (C.A.); 2 St. Mary Children’s Emergency Hospital, 700309 Iași, Romania; 3 “Nicolae Testemițanu” State University of Medicine and Pharmacy, MD-2004 Chișinău, Moldova; ninel.revenco@usmf.md; 4 Institute of Mother and Child, 93 Burebista Str., MD-2062 Chișinău, Moldova; 5 Clinical Rehabilitation Hospital, 700661 Iași, Romania; 6 “Cuza Vodă” Clinical Hospital of Obstetrics and Gynaecology, 700038 Iași, Romania

**Keywords:** juvenile idiopathic arthritis, autoimmune comorbidities, diagnostic spectrum, HLA-B27, enthesitis-related arthritis, autoimmune thyroiditis, paediatric rheumatology

## Abstract

**Background/Objectives:** Children with juvenile idiopathic arthritis (JIA) frequently develop additional autoimmune conditions during follow-up, yet the clinical and diagnostic profile of this comorbid subgroup is incompletely characterised in single-centre paediatric series. We aimed to describe the prevalence, clinical pattern, and diagnostic features of autoimmune comorbidities in a seven-year cohort of children with JIA monitored at a single tertiary paediatric centre, and to document the diagnostic protocols applied for each comorbidity. **Methods:** We conducted a retrospective descriptive observational study of 103 consecutive children with JIA classified according to the ILAR 2001 criteria and monitored at the Paediatric Rheumatology Unit of St. Mary Children’s Emergency Hospital, Iași, Romania, between 2017 and 2023, with the year 2020 excluded by design owing to the institutional reorganisation during the early COVID-19 pandemic. Autoimmune comorbidities were ascertained from medical records using ICD-10 coding and confirmed by subspecialty evaluation. The diagnostic approach for each comorbidity is reported in detail. Continuous variables are described using mean ± standard deviation, and categorical variables as frequencies (%). No inferential analysis was performed on predictor variables; the study is exploratory and hypothesis-generating. **Results:** Autoimmune comorbidity was identified in 21 of 103 children (20.4%; 95% confidence interval [CI] 13.1–29.5%), the JIA-AID subgroup. Patients were predominantly female (17 of 21, 81.0%) and aged over 12 years (10 of 21, 47.6%). Autoimmune thyroiditis was the most frequent comorbidity, present in 10 of 21 cases (47.6%) when the euthyroid, hypothyroid, and vitiligo-associated forms were combined, followed by inflammatory bowel disease (4 of 21, 19.0%), alopecia areata (4 of 21, 19.0%), localised scleroderma (2 of 21, 9.5%), and coeliac disease (1 of 21, 4.8%). Three patients (14.3%) had polyautoimmunity, defined as two or more autoimmune diagnoses in addition to JIA. The HLA-B27-positive enthesitis-related arthritis subtype, although small in absolute numbers, was over-represented within the JIA-AID subgroup: 5 of 7 HLA-B27-positive ERA patients (71.4%; 95% CI 29.0–96.3%) carried a coexisting autoimmune diagnosis, compared with 16 of 96 patients in the remainder of the cohort (16.7%; 95% CI 9.8–25.6%); the predominant comorbidity in this subtype was inflammatory bowel disease. **Conclusions:** Autoimmune comorbidity affected approximately one in five children with JIA in this single-centre cohort, with autoimmune thyroiditis and inflammatory bowel disease as the most frequent associations and a notable concentration of comorbidity within the HLA-B27-positive enthesitis-related arthritis subtype. These descriptive observations are hypothesis-generating and support the case for proactive multidisciplinary screening in selected subgroups. Prospective registry-based studies with explicit exposure classification and standardised functional outcomes will be needed to confirm the patterns reported here.

## 1. Introduction

Juvenile idiopathic arthritis (JIA) is the most common chronic rheumatic disease of childhood. The reported incidence ranges from 1.6 to 23 per 100,000 children, and the prevalence from 3.8 to 400 per 100,000, depending on geographical region and diagnostic ascertainment [1]. Under the International League of Associations for Rheumatology (ILAR) classification, the disease comprises seven heterogeneous categories—oligoarticular, rheumatoid factor (RF)-positive and RF-negative polyarticular, systemic, enthesitis-related arthritis (ERA), psoriatic, and undifferentiated arthritis—each with a distinct trajectory of joint inflammation, structural damage, and functional impairment [1,2].

A substantial body of evidence has positioned JIA within a broader autoimmune spectrum. Adults with a paediatric-onset history have been reported to carry a 37-fold higher risk of developing at least one additional autoimmune disease compared with the general population, with susceptibility windows around 3–5 and 10–15 years of age [3]. Cross-sectional cohorts indicate that 15–20% of children with JIA carry an additional autoimmune diagnosis at the time of evaluation [2]. The most frequently reported associations include autoimmune thyroiditis, inflammatory bowel disease (IBD), coeliac disease, alopecia areata, vitiligo, and localised scleroderma. The prevalence and distribution of these comorbidities, however, vary substantially between cohorts. The contribution of single-centre observational data to this body of evidence lies primarily in characterising the local clinical and diagnostic profile, identifying patient subgroups in which the comorbidity is concentrated, and informing the practical diagnostic algorithms applied in daily care.

The specific autoimmune comorbidities investigated in the present study each carry a distinct epidemiological signature within paediatric rheumatic disease. Autoimmune thyroid disease is the most consistently reported association: cross-sectional and inception-cohort data place its prevalence in JIA at 5–17%, with a clear preponderance in females, in ANA-positive patients, and in older age groups [2,4,5]. Inflammatory bowel disease is uncommon in unselected JIA cohorts but is strongly concentrated within the HLA-B27-positive enthesitis-related arthritis subtype, reflecting a shared axial spondyloarthropathy background [6,7,8,9,10]. Coeliac disease has been the subject of dedicated paediatric investigation: a systematic review positioned it as a recognised comorbidity of juvenile idiopathic arthritis and other paediatric rheumatic disorders [11], and a large population-based cohort has since documented a bidirectional association, with children with coeliac disease carrying an increased risk of subsequent JIA and joint complaints being a frequent presenting feature of coeliac disease itself [12,13]. Alopecia areata and vitiligo occur predominantly within the polyarticular and systemic subtypes and typically co-segregate with thyroid autoimmunity, forming a recognisable mucocutaneous–endocrine cluster [14,15,16,17]. Localised scleroderma, although rare, shares antinuclear seropositivity and profibrotic cytokine networks with juvenile idiopathic arthritis [18,19,20,21,22]. Familial autoimmunity is a further recurrent feature: registry data indicate that a substantial proportion of children with JIA have a first- or second-degree relative affected by an autoimmune condition, particularly within ANA-positive and female-predominant subsets [23,24].

From a diagnostic standpoint, autoimmune comorbidities in JIA present several specific challenges. First, the clinical presentation is often subclinical or oligosymptomatic—autoimmune thyroiditis, for instance, is most frequently detected on laboratory screening rather than on clinical examination [2,4]. Second, the diagnostic approach must be tailored to the comorbidity in question, integrating serological, endoscopic, dermatological, and genetic investigations under multidisciplinary coordination. Third, the temporal relationship between the JIA diagnosis and the comorbid autoimmune disorder is heterogeneous; some comorbidities precede the joint disease, others co-occur, and others develop several years into follow-up. Together these considerations argue for an explicit description of how autoimmune comorbidities are identified and confirmed at the bedside, rather than merely tabulated.

Against this background, we present a descriptive single-centre observational study of children with JIA followed for seven years at a Romanian tertiary paediatric centre. The aim of the study is threefold: (i) to describe the prevalence and clinical pattern of autoimmune comorbidities; (ii) to document, in detail, the diagnostic protocols applied for each comorbidity; and (iii) to characterise the subgroup of children with JIA and autoimmune comorbidity at the level of demographics, ILAR subtype, immunoserology, and therapeutic regimen. The study is explicitly descriptive and exploratory; no inferential analysis is performed, and we do not seek to identify independent predictors or risk factors. The observations are intended to be hypothesis-generating and to inform the design of larger prospective studies.

## 2. Materials and Methods

### 2.1. Study Design and Population

We conducted a retrospective descriptive observational study of 103 consecutive children diagnosed with JIA and monitored at the Paediatric Rheumatology Unit of St. Mary Children’s Emergency Hospital, Iași, Romania, between 1 January 2017 and 31 December 2023. The pandemic year 2020 was excluded by design, since the institution functioned as a regional COVID-19 referral centre during that year and the number of children with JIA admitted in that interval was insufficient for descriptive analysis. Patients reaching the upper paediatric age limit during follow-up continued evaluation at the Second Rheumatology Department of the Clinical Rehabilitation Hospital, Iași, which allowed for joint paediatric–adult monitoring during the transition window.

The study was conducted in accordance with the Declaration of Helsinki and was approved by the institutional Ethics Committee. As this was a retrospective observational study based exclusively on archived medical records, all data were anonymised at the source and no prospective contact with patients or their guardians was undertaken. The institutional Ethics Committee approved the use of the archived clinical data for the specific aims of this study without requiring additional individual consent, in recognition of the retrospective and non-interventional design. The manuscript follows the STROBE reporting recommendations for observational research.

### 2.2. Eligibility Criteria and Definitions

Eligible participants were aged ≤ 18 years and met the ILAR 2001 classification criteria for JIA [1], encompassing the oligoarticular, polyarticular RF-positive, polyarticular RF-negative, systemic, enthesitis-related, psoriatic, and undifferentiated subtypes. Patients with incomplete records or diagnoses subsequently reclassified as non-JIA inflammatory arthropathies were excluded.

The associated autoimmune conditions of interest were defined a priori on the basis of International Classification of Diseases, 10th Revision (ICD-10) coding and confirmed by the corresponding subspecialty evaluation. They comprised autoimmune thyroiditis (with normal thyroid function or with hypothyroidism), inflammatory bowel disease (IBD), coeliac disease, alopecia areata, vitiligo, and localised scleroderma (morphea). The composite category JIA-AID was used throughout the manuscript to denote a JIA patient with at least one of these autoimmune comorbidities at any time during follow-up. Polyautoimmunity was defined as the coexistence of two or more autoimmune diagnoses in addition to JIA. Chronic anterior uveitis was not included among the comorbidities of interest, as it is conventionally regarded as an extra-articular manifestation of JIA rather than an independent autoimmune diagnosis, and is already covered by established ophthalmological screening recommendations; this restriction of scope is revisited in Section 4.7.

### 2.3. Variables Collected

The following information was extracted from medical records for each patient: age at JIA diagnosis, sex, ILAR subtype, antinuclear antibody (ANA) status, RF status, HLA-B27 genotyping, family history of autoimmune disease in first- and second-degree relatives, the type and sequence of disease-modifying therapy (synthetic disease-modifying antirheumatic drugs [DMARDs], biological agents, systemic corticosteroids), and the documented status of disease activity at the most recent evaluation, classified by the treating rheumatologist as therapeutically controlled, partially controlled, or uncontrolled on the basis of clinical examination, inflammatory markers, and ongoing medication needs. Standardised composite disease-activity indices such as the Juvenile Arthritis Disease Activity Score (JADAS-27) or the Childhood Health Assessment Questionnaire (CHAQ) were not consistently available across the earliest pre-pandemic patients; we therefore report the categorical disease-control status descriptively, without using it as an outcome variable in any comparative analysis. Family history was recorded as reported by the guardians and included both organ-specific autoimmune disorders (autoimmune thyroid disease, type 1 diabetes mellitus, coeliac disease, vitiligo) and systemic autoimmune diseases (rheumatoid arthritis, systemic lupus erythematosus, and other connective-tissue disorders).

### 2.4. Diagnostic Protocols for Autoimmune Comorbidities

For each autoimmune comorbidity, the diagnostic workflow followed a sequential, multidisciplinary algorithm: a defined clinical or laboratory trigger initiated the workup, followed by initial screening in the rheumatology unit, confirmatory testing by the relevant subspecialty service, and registration of the final diagnosis with the corresponding ICD-10 code. Screening intensity was adapted to the ILAR subtype and the clinical phenotype. Only the elements specific to our institutional practice are detailed below; the broader diagnostic frameworks followed established international guidelines and are referenced accordingly.

Autoimmune thyroiditis. Screening was performed at the time of JIA diagnosis and repeated annually thereafter, or earlier in the presence of clinical or laboratory triggers. The institutional panel comprised free triiodothyronine (fT3), free thyroxine (fT4), and thyroid-stimulating hormone (TSH), together with anti-thyroid peroxidase (anti-TPO; cut-off 1–16 IU/mL), anti-thyroglobulin (anti-Tg; age-specific cut-offs of <38 IU/mL for children aged 1–11 years and <64 IU/mL for those aged 11–18 years), and anti-TSH receptor antibodies (>0.3 IU/L). TSH receptor antibodies were included as part of the standard institutional autoimmune thyroid panel for completeness of differential characterisation, but the diagnosis of chronic autoimmune (Hashimoto-type) thyroiditis was based on positive anti-TPO and/or anti-Tg together with characteristic ultrasonographic findings; no case in this cohort was diagnosed on the basis of TRAb positivity alone. High-frequency thyroid ultrasonography was performed in patients with abnormal function or positive autoantibodies, and confirmed cases were referred to paediatric endocrinology.

Alopecia areata. Cases were identified by clinical observation of patchy non-scarring hair loss and confirmed by dermoscopy (exclamation-mark hairs, yellow and black dots, tapered hairs). Extent of involvement was quantified with the Severity of Alopecia Tool (SALT) score [25]. In diagnostically uncertain cases a 4 mm punch biopsy was obtained. Coexisting autoimmunity was screened with an ANA-44 immunodot panel and thyroid autoantibodies. Confirmed cases were co-managed by dermatology and rheumatology.

Localised scleroderma (morphea). Suspicion was raised by the appearance of circumscribed indurated plaques with characteristic peripheral erythema. Confirmation required full-thickness skin biopsy at the active border of the lesion, with histopathology demonstrating dermal fibrosis and lymphoplasmacytic infiltrate. Lesional activity was assessed with the modified Localised Scleroderma Skin Severity Index (mLoSSI) [26]. Serological workup included ANA testing (indirect immunofluorescence on Hep-2 cells) and an ANA-44 immunodot panel with anti-Scl-70, anti-centromere, anti-RNA polymerase III, and anti-PM/Scl reactivities. Patients were followed up at six-month intervals to monitor lesional activity and screen for extracutaneous features.

Inflammatory bowel disease. Screening was triggered by gastrointestinal symptoms or by systemic features suggestive of intestinal inflammation (growth deceleration, refractory iron-deficiency anaemia, unexplained sustained elevation of inflammatory markers). A lower threshold for evaluation was applied to HLA-B27-positive enthesitis-related arthritis. The initial workup comprised faecal calprotectin (>150 µg/g considered indicative of intestinal inflammation in the paediatric age range [27]), C-reactive protein, complete blood count, serum albumin, and an inflammatory bowel disease autoantibody profile (ASCA IgA/IgG, pANCA). Confirmatory evaluation followed the ESPGHAN revised Porto criteria [28], comprising upper gastrointestinal endoscopy and ileocolonoscopy with segmental biopsies and small-bowel imaging.

Coeliac disease. Serological screening was undertaken in patients with suggestive gastrointestinal symptoms, growth deceleration, unexplained iron-deficiency anaemia, or an established autoimmune comorbidity. The diagnostic pathway followed ESPGHAN guidelines [29]: total serum IgA was measured first (to exclude selective IgA deficiency), followed by anti-tissue transglutaminase IgA (cut-off ≥ 10 U/mL by FEIA or ELISA), confirmed by endomysium IgA antibodies (titre ≥ 1/10). HLA-DQ2/DQ8 genotyping was performed in atypical cases. Duodenal biopsy was performed where the non-biopsy criterion was not fulfilled, with histopathological grading by the modified Marsh–Oberhüber classification [30].

Vitiligo. The diagnosis was made clinically by a paediatric dermatologist on the basis of acquired depigmented macules, supported by Wood-lamp examination. Lesional distribution was classified as segmental, non-segmental, or mixed. Given the strong co-segregation of vitiligo with thyroid autoimmunity, all cases underwent thyroid function testing and anti-thyroid antibody screening at diagnosis and annually thereafter, together with an ANA-44 immunodot panel. Skin biopsy was reserved for atypical presentations.

Documentation and case ascertainment. For every confirmed comorbidity, the diagnosis was registered in the patient’s medical record under the corresponding ICD-10 code, together with the date of diagnosis, the responsible subspecialty service, the diagnostic modality used (clinical, serological, imaging, or histopathological), and the therapeutic plan. Case ascertainment for the present study was performed by cross-checking ICD-10 codes recorded in the rheumatology and the relevant subspecialty databases against the original clinical notes, to minimise the risk of misclassification and to ensure that each reported comorbidity was supported by a documented diagnostic workflow.

### 2.5. Pandemic Period Documentation

The cohort was stratified into a pre-pandemic interval (2017–2019) and a post-pandemic interval (2021–2023) for purely descriptive temporal characterisation. Documented SARS-CoV-2 infection during follow-up was recorded when available, on the basis of real-time reverse-transcription polymerase chain reaction (RT-PCR) testing of nasopharyngeal swabs performed in the institutional molecular biology laboratory with a CE-IVD-approved multiplex RT-PCR assay (with cycle threshold cut-off of 40 for positivity) and, where serological confirmation was performed, by quantitative anti-SARS-CoV-2 IgG and IgM antibody titres measured by the microblot method according to the manufacturer’s specifications. SARS-CoV-2 status was not systematically ascertained across the entire cohort; for this reason, SARS-CoV-2 exposure is not used as an analytic variable in this study, and the pre- and post-pandemic strata are presented purely as descriptive temporal categories.

### 2.6. Statistical Approach

Data were collected in Microsoft Excel (Microsoft Corp., Redmond, WA, USA) and described using IBM SPSS Statistics version 22.0 (IBM Corp., Armonk, NY, USA). Continuous variables are reported as mean ± standard deviation, and categorical variables as frequencies (%). Subgroup characteristics are presented descriptively for the JIA-AID and non-AID groups and for the pre- and post-pandemic strata. No inferential statistical analysis (logistic regression, hypothesis testing on predictor variables, or estimation of odds ratios) was performed, as the study is explicitly descriptive and the event count and design do not support stable inferential modelling. The findings are intended to be exploratory and hypothesis-generating. Exact 95% confidence intervals (Clopper–Pearson method) are reported for descriptive proportions derived from subgroups with small denominators, in order to quantify the uncertainty around point estimates without invoking inferential hypothesis testing.

## 3. Results

### 3.1. Cohort Characteristics

The study cohort comprised 103 children with JIA followed across two non-overlapping intervals: 68 patients (66.0%) in the pre-pandemic period (2017–2019) and 35 patients (34.0%) in the post-pandemic period (2021–2023). The age at JIA diagnosis fell predominantly within the 6–12-year window (*n* = 48; 46.6%), followed by the >12-year category (*n* = 41; 39.8%); only 14 children (13.6%) were younger than 6 years at diagnosis. Female patients accounted for 64 of 103 children (62.1%). The distribution of ILAR subtypes was, in order of frequency, RF-negative polyarticular JIA (*n* = 22; 21.4%), ANA-negative extended oligoarticular JIA (*n* = 19; 18.4%), ANA-positive extended oligoarticular JIA (*n* = 9; 8.7%), RF-positive polyarticular JIA (*n* = 12; 11.7%), psoriatic JIA (*n* = 11; 10.7%), oligoarticular JIA (*n* = 10; 9.7%), enthesitis-related arthritis (*n* = 15 combined, of which 7 HLA-B27-positive and 8 HLA-B27-negative), and systemic JIA (*n* = 5; 4.9%).

A family history of autoimmune disease was documented in 89 of 103 patients (86.4%): 75 patients (72.8%) reported one affected first- or second-degree relative, and 14 patients (13.6%) reported two or more (Table 1). Disease control at the most recent evaluation was classified as therapeutically controlled in 65 patients (63.1%), partially controlled in 22 (21.4%), and uncontrolled in 16 (15.5%). With respect to disease-modifying therapy, conventional synthetic DMARD monotherapy was used in 49 of 103 patients (47.6%) and biological therapy in 45 (43.7%); the remaining 9 patients (8.7%) had not yet initiated disease-modifying therapy at the time of evaluation. Systemic corticosteroids were used as a temporary add-on, in combination with the underlying DMARD or biological treatment, in 10 cases (9.7%), all among children without autoimmune comorbidity.

### 3.2. Spectrum of Autoimmune Comorbidities

Autoimmune comorbidity was identified in 21 of 103 children (20.4%; 95% CI 13.1–29.5%). The JIA-AID subgroup was predominantly female (17 of 21, 81.0% versus 47 of 82, 57.3% in the non-AID group) and was distributed across the older age categories: 10 patients (47.6%) were aged over 12 years, 9 (42.9%) were aged 6–12 years, and only 2 (9.5%) were younger than 6 years at the time of JIA diagnosis. Three patients (14.3% of the JIA-AID subgroup) presented with polyautoimmunity, defined as two or more autoimmune diagnoses additional to JIA; all three carried the vitiligo–autoimmune thyroiditis cluster (Supplementary Table S1).

Autoimmune thyroiditis was the most frequent comorbidity. When all forms were combined (overt hypothyroidism, subclinical/euthyroid, and the vitiligo-associated form), autoimmune thyroiditis was present in 10 of 21 JIA-AID patients (47.6%): three with overt hypothyroidism (14.3%), four with the subclinical euthyroid form (19.0%), and three associated with vitiligo (14.3%). Inflammatory bowel disease affected 4 of 21 JIA-AID patients (19.0%); three were Crohn-type and one had an indeterminate presentation, all confirmed histologically. Alopecia areata affected 4 of 21 patients (19.0%; 95% CI 5.4–41.9%), distributed across psoriatic (*n* = 2), RF-negative polyarticular (*n* = 1), and systemic (*n* = 1) JIA. Localised scleroderma (morphea) was observed in 2 of 21 patients (9.5%), both with a single circumscribed plaque type. Coeliac disease was diagnosed in 1 of 21 patients (4.8%). Vitiligo in our cohort occurred exclusively in association with autoimmune thyroiditis (3 of 21, 14.3%) and was not observed in isolation.

A case-by-case description of the 21 JIA-AID patients, including sex, age category, ILAR subtype, autoimmune diagnosis, period of follow-up, documented SARS-CoV-2 status, and therapeutic regimen, is provided in Supplementary Table S1.

### 3.3. Clinical Distribution by JIA Subtype

The distribution of autoimmune comorbidity across ILAR subtypes was uneven (Table 1 and Table 2). The HLA-B27–positive enthesitis-related arthritis subtype was the most strikingly affected: 5 of 7 HLA-B27–positive ERA patients (71.4%; 95% CI 29.0–96.3%) carried a coexisting autoimmune diagnosis, compared with 16 of 96 patients in the remainder of the cohort (16.7%; 95% CI 9.8–25.6%). The predominant comorbidity in the HLA-B27–positive ERA subtype was inflammatory bowel disease, which accounted for three of the five comorbid cases in this subtype; the remaining two cases comprised one autoimmune thyroiditis and one vitiligo–thyroiditis association.

The ANA-positive extended oligoarticular subtype (eoJIA-ANA+) contributed 4 of the 9 patients in this subtype (44.4%) to the JIA-AID subgroup, mostly with autoimmune thyroiditis and vitiligo-associated thyroiditis. Psoriatic JIA contributed 4 of 11 patients (36.4%), with a clinical mixture of inflammatory bowel disease, autoimmune thyroiditis, and alopecia areata. The remaining JIA-AID cases were distributed across RF-positive polyarticular JIA (2 of 12, 16.7%), RF-negative polyarticular JIA (4 of 22, 18.2%), and systemic JIA (2 of 5, 40.0%). The oligoarticular, ANA-negative extended oligoarticular, and HLA-B27-negative ERA subtypes contributed no JIA-AID cases (Table 1).

### 3.4. Therapeutic Regimen in the JIA-AID Subgroup

Within the JIA-AID subgroup, biological therapy was the predominant disease-modifying treatment (17 of 21, 81.0%), reflecting the heavier disease burden and the more aggressive ILAR subtype distribution of these patients. Conventional synthetic DMARD monotherapy was used in 4 of 21 cases (19.0%), all in patients with milder disease at presentation. No JIA-AID patient required maintenance corticosteroid co-therapy. Full therapeutic control at last evaluation was documented in 9 of 21 (42.9%) JIA-AID patients, partial control in 7 (33.3%), and uncontrolled disease in 5 (23.8%). These descriptive proportions are reported without inferential comparison with the non-AID subgroup, in accordance with the descriptive design of the study.

### 3.5. Pre- and Post-Pandemic Descriptive Distribution

The temporal distribution of JIA-AID cases across the two calendar intervals is summarised in Supplementary Table S1. Documented SARS-CoV-2 infection during follow-up was recorded for six patients within the JIA-AID subgroup; SARS-CoV-2 status was not systematically ascertained for the remainder of the cohort and is therefore not used as an analytic variable. As detailed in Section 4.7 (Limitations), the unequal person-time at risk between the two intervals, combined with the institutional reorganisation during 2020 and the absence of systematic SARS-CoV-2 ascertainment, precludes any meaningful comparison of prevalence or incidence between the pre- and post-pandemic strata. The descriptive distribution is presented here for transparency only.

## 4. Discussion

The cohort reported here contributes to a small but informative body of descriptive literature on the autoimmune burden of paediatric JIA, from a vantage point that is, to our knowledge, novel in the Romanian context: a seven-year monitoring window at a single tertiary paediatric centre. We deliberately frame the manuscript as descriptive: the design does not support causal or inferential claims, and we therefore present observations as the basis for future hypothesis-driven studies rather than as definitive risk estimates.

### 4.1. Magnitude and Pattern of Autoimmune Comorbidity

One in five children in our cohort carried at least one additional autoimmune diagnosis, a figure consistent with the 15–20% range described by Tronconi and colleagues and by the multicentre analysis of Lovell and colleagues [2,3]. The marked female predominance within the JIA-AID subgroup (81%) and the over-representation of children older than 12 years align with the age- and sex-specific windows of autoimmune susceptibility reported in larger paediatric series [31]. The descriptive prevalence of polyautoimmunity (14.3% of the JIA-AID subgroup, all carrying the vitiligo–thyroiditis cluster) parallels the observation that mucocutaneous and endocrine autoimmunity tend to co-segregate within affected children.

Autoimmune thyroiditis was the most frequent associated condition. When all forms—overt, subclinical, and vitiligo-associated—were combined, thyroid involvement reached 47.6% of the JIA-AID subgroup. This figure is towards the higher end of the spectrum reported in the literature, where thyroid involvement is estimated at 5–17% in unselected JIA cohorts and rises to nearly one in five in ERA-enriched series [2,4,32]. Most cases in our cohort were subclinical, mirroring the literature in which autoantibody positivity precedes overt dysfunction by months to years [4,33]. Inflammatory bowel disease and alopecia areata followed in descriptive frequency, with localised scleroderma and coeliac disease at the lower end.

The comparative frequency of the different autoimmune comorbidities observed in our cohort should be interpreted in the context of the differential intensity of surveillance applied to each condition. Autoimmune thyroiditis was investigated by a universal, protocol-driven annual screening in every patient (thyroid function and thyroid autoantibodies), whereas inflammatory bowel disease and coeliac disease were assessed only when a clinical or laboratory trigger was present. Under this asymmetric surveillance strategy, the higher observed frequency of thyroiditis relative to the gastrointestinal comorbidities probably reflects, at least in part, the greater capacity of a universal annual screen to detect oligosymptomatic and subclinical disease, rather than a truly greater underlying biological frequency. The magnitude of this ascertainment bias cannot be quantified from our data, but its direction is consistent across the cohort: the reported proportion of thyroiditis is likely to represent the true prevalence more closely than the reported proportions of IBD or coeliac disease, which should be regarded as minimum estimates. This point is further discussed in Section 4.7 as a formal limitation of the design.

### 4.2. The HLA-B27-Positive ERA Subtype: A Distinctive Clinical Cluster

The most striking observation in our descriptive analysis is the concentration of autoimmune comorbidity within the HLA-B27-positive ERA subtype. Five of seven HLA-B27-positive ERA patients (71.4%; 95% CI 29.0–96.3%) carried a coexisting autoimmune diagnosis, predominantly inflammatory bowel disease (three of the five comorbid cases). In the remainder of the cohort, the comorbidity prevalence was 16 of 96 patients (16.7%; 95% CI 9.8–25.6%). We emphasise that the small absolute number of HLA-B27-positive ERA patients limits the precision of this observation, and we do not present an inferential risk estimate; however, the pattern is biologically coherent and warrants attention.

The mechanistic plausibility of this clustering is well established. The gut–joint axis in HLA-B27-associated spondyloarthropathy provides a unified immunopathological substrate that links subclinical gut inflammation, dysbiosis, and entheseal stress responses, with the IL-23/IL-17 cytokine axis at the centre of the shared inflammatory programme [6,7,8,9,10]. Subclinical ileitis is detectable on endoscopy in a substantial fraction of adults with axial spondyloarthritis, and the bidirectional clinical relationship between paediatric ERA and IBD is well described in larger registries [6,7,8,9,10].

From a diagnostic standpoint, the practical implication is that children diagnosed with HLA-B27-positive ERA warrant an explicit search for gastrointestinal symptoms (chronic abdominal pain, growth deceleration, persistent fatigue, anaemia disproportionate to disease activity, faecal calprotectin elevation) and a low threshold for gastroenterological evaluation, regardless of the activity status of the joint disease. This recommendation is consistent with the descriptive concentration we observed and with the broader literature; it is not, however, derived from inferential analysis in our dataset.

### 4.3. Comorbidity-Specific Considerations

Autoimmune thyroiditis, mostly subclinical, was the most prevalent comorbidity. The cross-talk between Hashimoto’s thyroiditis and JIA reflects shared HLA susceptibility alleles, a female predisposition, and overlapping autoantibody profiles [4,33,34,35]. Sapountzi and colleagues reported Hashimoto’s thyroiditis in 16.9% of JIA patients, mostly subclinical, with higher rates in female patients (80%) and the ERA subtype (72.7%) [4]. Tronconi and colleagues found thyroid involvement in 10.1% of JIA patients, again most commonly without overt dysfunction and in a female-predominant 4:1 distribution, with a family history of autoimmunity in 70% of these cases and familial Hashimoto’s thyroiditis in 30.8% [2]. Autoimmune hyperthyroidism is rarely reported in JIA [33]. The diagnostic implication is that periodic thyroid autoantibody screening combined with thyroid function testing represents a low-cost, high-yield strategy for the early identification of subclinical disease in selected paediatric JIA subgroups.

Vitiligo in our cohort occurred exclusively in association with autoimmune thyroiditis, consistent with reports linking vitiligo to thyroid disorders and JIA through shared genetic markers such as PTPN22 mutations [14,15]. The JIA–vitiligo association is rare in unselected cohorts (1.1% in Haşlak and colleagues) and most commonly co-occurs with thyroid autoimmunity rather than as an isolated cutaneous comorbidity [14,36,37].

Localised scleroderma (morphea) was observed in 9.5% of JIA-AID cases. The mechanistic overlap with JIA reflects shared profibrotic cytokine networks and antinuclear seropositivity [18,19,20,21,22]. Although associations with methotrexate and anti-TNF therapy have been reported anecdotally, no patients in our cohort progressed to systemic scleroderma or lost JIA control as a consequence of the cutaneous comorbidity.

Inflammatory bowel disease affected nearly one in five JIA-AID patients in our descriptive cohort, with the overlap most pronounced in the HLA-B27-positive ERA subtype, in line with the 20- to 40-fold higher IBD risk reported in JIA compared with the general paediatric population [6,7,8,9,10]. The shared immunopathology involves the IL-23/IL-17 axis, gut dysbiosis, and a common HLA susceptibility background. Faecal calprotectin combined with paediatric-appropriate endoscopic protocols remains the diagnostic standard.

Coeliac disease was identified in a single patient of the JIA-AID subgroup (4.8%; 95% CI 0.1–23.8%), a proportion that is consistent with the modest but reproducible association reported in the paediatric literature. A dedicated review of the topic positions coeliac disease as a recognised comorbidity within juvenile idiopathic arthritis and other paediatric rheumatic disorders, and highlights the practical challenge that the two conditions share several non-specific presenting features, including growth deceleration, iron-deficiency anaemia, and generalised musculoskeletal complaints [11]. The association is bidirectional: a recent nationwide population-based cohort study has documented an increased risk of juvenile idiopathic arthritis and rheumatoid arthritis in individuals with coeliac disease, with the hazard peaking in the first years after the coeliac disease diagnosis [12]. From the opposite direction, a recent systematic review and meta-analysis reports that joint complaints are frequent presenting or accompanying features of coeliac disease itself, further reinforcing the recommendation that a low threshold for coeliac serology should apply in children with unexplained polyarticular or oligoarticular complaints [13]. The low prevalence observed in our cohort should be interpreted cautiously in view of the surveillance asymmetry noted above: coeliac serology was undertaken on clinical or laboratory indication rather than as a universal annual screen, and the reported proportion should be regarded as a minimum estimate of the true underlying frequency.

Alopecia areata (19.0% of JIA-AID cases, observed in the psoriatic, polyarticular, and systemic subtypes) has a smaller direct impact on rheumatic disease control but remains clinically relevant as part of broader autoimmune surveillance. A polyarticular subtype-specific predisposition to alopecia areata has been described and may reflect shared HLA susceptibility [16,17].

### 4.4. Practical Screening Recommendations

Several pragmatic recommendations emerge from the descriptive pattern of comorbidity in our cohort. These are framed as suggestions for diagnostic practice rather than as evidence-based screening guidelines, and they require validation in prospective cohorts before adoption as standards.

On the basis of the descriptive pattern observed in the present cohort and its concordance with the broader paediatric rheumatology literature, we propose the following screening strategy as expert opinion, to be validated in prospective cohorts. For thyroid autoimmunity, thyroid function testing and thyroid autoantibody measurement should be obtained at the time of JIA diagnosis and repeated annually thereafter in every patient, irrespective of ILAR subtype; the yield is highest in female, ANA-positive, and polyarticular patients, and in those over the age of 12 years. For inflammatory bowel disease, faecal calprotectin should be measured at JIA diagnosis in all children with HLA-B27-positive enthesitis-related arthritis and repeated at least annually or whenever gastrointestinal symptoms, growth deceleration, refractory iron-deficiency anaemia, or unexplained sustained elevation of inflammatory markers occur; in other JIA subtypes, calprotectin should be reserved for symptom-driven investigation. For coeliac disease, screening with total serum IgA and anti-tissue transglutaminase IgA should be performed at JIA diagnosis in every patient and reconsidered whenever an additional autoimmune diagnosis is made, given the well-documented co-segregation of coeliac disease with autoimmune thyroid disease. The identification of any single autoimmune comorbidity should in itself prompt a broader assessment for additional autoimmune phenomena, since these tend to co-segregate within affected children, as illustrated by the vitiligo–thyroiditis cluster in the present cohort. For cutaneous autoimmunity (alopecia areata, vitiligo, localised scleroderma), evaluation by a paediatric dermatologist is warranted whenever a new lesion is identified during routine clinical review, with concurrent thyroid function and thyroid autoantibody testing given the strong co-segregation described above. This screening strategy is presented as an expert-opinion proposal derived from a single-centre descriptive dataset and requires prospective validation before adoption as a standard-of-care recommendation. While international consensus documents on JIA management, including the 2024 EULAR/PReS recommendations for Still’s disease and adult-onset AOSD [38] and the 2022 ACR guideline on JIA therapeutic approaches [39], provide detailed pharmacological pathways, they do not address structured screening for autoimmune comorbidities in a granular way; the present recommendations are intended as a practical complement rather than as a replacement.

### 4.5. Treatment Intensity in the JIA-AID Subgroup

A notable observation in our cohort is the higher use of biological therapy within the JIA-AID subgroup compared with the remainder of the cohort (17 of 21 patients, 81.0%, versus 28 of 82, 34.1%). Several non-exclusive explanations should be considered. First, the JIA-AID subgroup is enriched in ILAR subtypes with an intrinsically greater disease burden and a higher frequency of biological therapy initiation, namely HLA-B27-positive enthesitis-related arthritis, ANA-positive extended oligoarticular JIA, polyarticular JIA, and psoriatic JIA, whereas the milder oligoarticular subtypes contributed no JIA-AID cases. Second, referral bias is likely: children with more complex disease, and particularly those with recognised or suspected additional autoimmunity, are preferentially referred to and retained at a tertiary paediatric rheumatology centre, which further concentrates severe phenotypes in the JIA-AID subgroup. Third, we cannot exclude the possibility that biological therapy itself contributes to a differential rate of comorbidity detection: children on biological agents are subject to more intensive laboratory monitoring, which increases the probability of identifying subclinical or oligosymptomatic autoimmune conditions. The descriptive design of the study does not allow us to disentangle these contributions, and no causal claim is made; the association is reported as a clinically relevant observation that warrants prospective evaluation.

### 4.6. Strengths

This study has several methodological strengths worth declaring. First, the seven-year monitoring window provides one of the longer single-centre observational records for autoimmune comorbidity in paediatric JIA reported from the eastern European region. Second, the diagnostic workup for each comorbidity followed standardised institutional protocols, integrating serological, ultrasonographic, endoscopic, and histopathological assessment under multidisciplinary coordination; this minimises the risk of misclassification of comorbidity status. Third, case ascertainment relied on ICD-10 coding cross-checked against the original subspecialty records, so that every reported comorbidity is supported by a documented diagnostic workflow; this reduces the risk of misclassification, although, as set out in Section 4.1 and Section 4.7, it does not offset the differential surveillance intensity applied across comorbidities. Fourth, the explicit case-by-case description of the 21 JIA-AID patients (Supplementary Table S1) provides the granularity required for cross-cohort comparison and for the design of subsequent prospective registry studies.

### 4.7. Limitations

This study has several limitations that we declare explicitly.

Design. The study is retrospective, single-centre, and descriptive. No grouped comparison or inferential analysis was performed on predictor variables, and we do not draw causal conclusions or identify risk factors. The descriptive proportions and the clinical patterns reported here should be interpreted as hypothesis-generating.

Selection bias. The cohort was assembled at a single tertiary paediatric centre and probably reflects more severe or treatment-resistant JIA phenotypes than the average paediatric rheumatology caseload. Children with milder disease followed in primary or secondary care are under-represented. The absence of a non-JIA paediatric control group means that we cannot quantify the additional autoimmune risk attributable to JIA itself, only the prevalence and distribution within JIA. Chronic anterior uveitis was excluded from the panel of comorbidities by design, so the present figures do not describe the total extra-articular burden of JIA in this cohort.

Information bias. Data extraction was retrospective and depended on the completeness and standardisation of paediatric rheumatology records over a seven-year interval that included a major change in clinical workflow during 2020–2021. Standardised composite disease-activity indices (JADAS-27, CHAQ, JAMAR) were not consistently recorded across the cohort and are therefore not reported.

Limited cohort size. With 21 JIA-AID cases distributed across multiple comorbidity categories and ILAR subtypes, our descriptive estimates have wide uncertainty, particularly within the smaller subtype-specific strata such as HLA-B27-positive ERA (*n* = 7). We have deliberately refrained from inferential analysis precisely because the design and event count do not support stable modelling.

Pandemic period limitations. SARS-CoV-2 exposure was not systematically ascertained across the entire cohort, person–time at risk was not balanced between the pre- and post-pandemic intervals, and the pandemic period overlapped with substantial changes in healthcare access, follow-up frequency, and screening intensity. For these reasons, the pre- and post-pandemic stratification is presented as a descriptive temporal characterisation rather than as a comparison of exposure to viral infection.

External validity. The cohort is drawn from a single geographical region (northeastern Romania) and a single referral centre; generalisability to populations with different ethnic, genetic, or healthcare-access profiles cannot be assumed without external replication.

Ascertainment and referral biases warrant explicit discussion. The cohort was assembled at a single tertiary paediatric rheumatology centre that acts as regional referral hub for the northeastern region of Romania, and probably contains an over-representation of severe, treatment-resistant, or diagnostically complex phenotypes compared with the paediatric rheumatology caseload of a secondary care setting. This referral pattern is likely to inflate the estimated prevalence of autoimmune comorbidity relative to a population-based paediatric JIA cohort. Furthermore, as noted in Section 4.1, the diagnostic workflow applied differential intensity across comorbidities, with universal annual screening for thyroid autoimmunity and symptom-triggered evaluation for gastrointestinal comorbidities. The reported comparative frequencies should therefore be interpreted as descriptive observations reflecting both underlying biological patterns and the local diagnostic infrastructure, rather than as unbiased population estimates.

The unavailability of the Juvenile Arthritis Disease Activity Score (JADAS-27), the Childhood Health Assessment Questionnaire (CHAQ), and the Juvenile Arthritis Multidimensional Assessment Report (JAMAR) across the entire follow-up period represents an important methodological limitation. Categorical clinician-assessed disease-control status was used as a surrogate for disease activity, but this cannot substitute for validated composite indices. The absence of standardised activity measures constrains the interpretation of our findings in two respects: first, we cannot rigorously test whether higher disease activity is associated with a greater probability of detecting autoimmune comorbidity; second, we cannot adjust the observed higher use of biological therapy in the JIA-AID subgroup for baseline disease severity, which limits the mechanistic interpretation of the treatment intensity pattern reported in Section 4.5. Prospective inclusion of JADAS and CHAQ measurements should be a priority for future cohorts investigating this question.

## 5. Conclusions

In this seven-year descriptive single-centre observational study, autoimmune comorbidity affected approximately one in five children with JIA. Autoimmune thyroiditis was the most frequent association (47.6% of the JIA-AID subgroup), followed by inflammatory bowel disease and alopecia areata. The HLA-B27-positive enthesitis-related arthritis subtype was over-represented within the comorbid subgroup, with five of seven patients (71.4%) carrying a coexisting autoimmune diagnosis, predominantly inflammatory bowel disease. Three patients (14.3% of the JIA-AID subgroup) presented with polyautoimmunity, all within the vitiligo–thyroiditis cluster.

These observations are descriptive and hypothesis-generating; we do not present them as evidence of causal risk or as validated screening recommendations. The pattern supports the case for proactive multidisciplinary diagnostic surveillance in selected subgroups, with particular attention to gastrointestinal evaluation in HLA-B27-positive ERA and to thyroid screening in female ANA-positive patients. Prospective registry-based studies with standardised functional outcomes, balanced exposure classification, and an appropriate control population will be needed to confirm the patterns reported here. The observation regarding the HLA-B27-positive enthesitis-related arthritis subgroup should be interpreted with caution, since it derives from a very small denominator (seven patients) and carries wide confidence limits (95% CI 29.0–96.3%); external replication in larger multicentre paediatric cohorts is required before this signal can inform clinical decision-making.

## Figures and Tables

**Table 1:** Characteristics of JIA patients with and without associated autoimmune diseases.

Parameters	Total (*n* = 103)	JIA-AID (*n* = 21; 20.4%)	Non-AID (*n* = 82; 79.6%)
**Study period**			
Pre-pandemic (2017–2019)	68 (66.0%)	15 (71.4%)	53 (64.6%)
Post-pandemic (2021–2023)	35 (34.0%)	6 (28.6%)	29 (35.4%)
**Age at JIA diagnosis**			
<6 years	14 (13.6%)	2 (9.5%)	12 (14.6%)
6–12 years	48 (46.6%)	9 (42.9%)	39 (47.6%)
>12 years	41 (39.8%)	10 (47.6%)	31 (37.8%)
**Sex**			
Male	39 (37.9%)	4 (19.0%)	35 (42.7%)
Female	64 (62.1%)	17 (81.0%)	47 (57.3%)
**Family history of autoimmune disease**			
Negative	14 (13.6%)	4 (19.1%)	10 (12.2%)
One affected relative	75 (72.8%)	13 (61.9%)	62 (75.6%)
Two or more affected relatives	14 (13.6%)	4 (19.0%)	10 (12.2%)
**JIA subtype (ILAR)**			
Oligoarticular (oJIA)	10 (9.7%)	0 (0.0%)	10 (12.2%)
Extended oligoarticular, ANA+ (eoJIA-ANA+)	9 (8.7%)	4 (19.1%)	5 (6.1%)
Extended oligoarticular, ANA− (eoJIA-ANA−)	19 (18.4%)	0 (0.0%)	19 (23.2%)
Polyarticular RF+ (RF+ pJIA)	12 (11.6%)	2 (9.5%)	10 (12.2%)
Polyarticular RF− (RF− pJIA)	22 (21.4%)	4 (19.0%)	18 (21.9%)
Systemic JIA	5 (4.9%)	2 (9.5%)	3 (3.7%)
ERA HLA-B27+	7 (6.8%)	5 (23.8%)	2 (2.4%)
ERA HLA-B27−	8 (7.8%)	0 (0.0%)	8 (9.8%)
Psoriatic JIA	11 (10.7%)	4 (19.1%)	7 (8.5%)
**Therapy at last evaluation**			
Biological therapy	45 (43.7%)	17 (81.0%)	28 (34.1%)
Synthetic DMARD monotherapy	49 (47.6%)	4 (19.0%)	45 (54.9%)
No disease-modifying therapy	9 (8.7%)	0 (0.0%)	9 (11.0%)
Corticosteroid add-on *	10 (9.7%)	0 (0.0%)	10 (12.2%)
**Disease control status**			
Therapeutically controlled	65 (63.1%)	9 (42.9%)	56 (68.3%)
Partially controlled	22 (21.4%)	7 (33.3%)	15 (18.3%)
Uncontrolled	16 (15.5%)	5 (23.8%)	11 (13.4%)

Categorical variables are presented as frequency (%). The table is descriptive; no inferential comparison was performed between the JIA-AID and non-AID groups. Disease-modifying therapy categories (biological therapy, synthetic DMARD monotherapy, no disease-modifying therapy) are mutually exclusive and together account for all 103 patients. * Corticosteroid add-on indicates short-term systemic corticosteroid administered in combination with the underlying disease-modifying treatment, and is therefore overlapping with the categories above. Abbreviations: ANA, antinuclear antibodies; DMARD, disease-modifying antirheumatic drug; eoJIA, extended oligoarticular JIA; ERA, enthesitis-related arthritis; JIA-AID, juvenile idiopathic arthritis with associated autoimmune disease; oJIA, oligoarticular JIA; pJIA, polyarticular JIA; RF, rheumatoid factor.

**Table 2:** Frequency of autoimmune comorbidities by JIA subtype.

JIA Subtype (ILAR)	Total Patients in Subtype	Patients with Autoimmune Comorbidity (*n*, %)	Comorbidity Distribution Within Subtype
ERA HLA-B27+	7	5 (71.4%)	Inflammatory bowel disease (3 of 5)
eoJIA-ANA+	9	4 (44.4%)	Autoimmune thyroiditis (1)/morphea (1)/coeliac disease (1)/vitiligo + thyroiditis (1)
Systemic JIA	5	2 (40.0%)	Alopecia areata (1)/vitiligo + thyroiditis (1)
Psoriatic JIA	11	4 (36.4%)	Alopecia areata (2)/IBD (1)/thyroiditis (1)
RF+ pJIA	12	2 (16.7%)	Autoimmune thyroiditis (2)
RF− pJIA	22	4 (18.2%)	Thyroiditis (2)/alopecia (1)/morphea (1)
Oligoarticular (oJIA)	10	0 (0.0%)	None
eoJIA-ANA−	19	0 (0.0%)	None
ERA HLA-B27−	8	0 (0.0%)	None

Descriptive frequencies of autoimmune comorbidity by JIA subtype. No inferential comparison or risk estimation is presented. Abbreviations as in Table 1.

## Data Availability

The original contributions presented in this study are included in the article/Supplementary Material. Further inquiries can be directed to the corresponding author.

## Supplementary Materials

The following supporting information can be downloaded at: https://www.mdpi.com/article/10.3390/diagnostics16152381/s1, Table S1. Case-by-case clinical and therapeutic profile of the 21 JIA-AID patients.
