# Supplementary material for: Clinical Profile and Diagnostic Spectrum of Autoimmune Comorbidities in Juvenile Idiopathic Arthritis: A Descriptive Single-Centre Observational Study
Source: Diagnostics (Basel). 2026 Jul 29;16(15):2381. doi: 10.3390/diagnostics16152381 (PMC13466197; doi:10.3390/diagnostics16152381)
Supplement: Supplementary file 1 [file diagnostics-16-02381-s001.zip › diagnostics-4419174-supplementary.pdf]

Supplementary Table S1

Clinical Profile and Diagnostic Spectrum of Autoimmune Comorbidities in Juvenile Idiopathic Arthritis: A Descriptive Single-Centre Observational Study

Table S1. Case-by-case clinical and therapeutic profile of the 21 JIA-AID patients.

Case	Sex	Age	JIA subtype	Comorbidity	Period	COVID	Therapy
**1**	M	6–12 y	ERA HLA-B27+	AIT, hypothyroid	Pre	No	Biological
**2**	F	6–12 y	RF+ pJIA	AIT, hypothyroid	Post	Yes	Biological
**3**	F	> 12 y	RF+ pJIA	AIT, hypothyroid	Post	Yes	Biological
**4**	F	6–12 y	eoJIA-ANA+	AIT, euthyroid	Pre	No	Biological
**5**	F	6–12 y	eoJIA-ANA+	Coeliac disease	Pre	No	Biological
**6**	F	6–12 y	RF− pJIA	AIT, euthyroid	Post	Yes	Biological
**7**	F	6–12 y	RF− pJIA	AIT, euthyroid	Post	Yes	Biological
**8**	F	> 12 y	RF− pJIA	Morphea	Pre	No	Biological
**9**	F	< 6 y	eoJIA-ANA+	Morphea	Post	Yes	csDMARDs
**10**	F	> 12 y	Psoriatic JIA	IBD	Pre	No	Biological
**11**	M	> 12 y	ERA HLA-B27+	IBD	Pre	No	Biological
**12**	M	> 12 y	ERA HLA-B27+	IBD	Pre	No	Biological
**13**	F	> 12 y	ERA HLA-B27+	IBD	Post	Yes	csDMARDs
**14**	F	6–12 y	Psoriatic JIA	AIT, euthyroid	Pre	No	Biological
**15**	F	> 12 y	Psoriatic JIA	Alopecia areata	Pre	No	csDMARDs
**16**	F	> 12 y	Psoriatic JIA	Alopecia areata	Pre	No	csDMARDs
**17**	F	6–12 y	RF− pJIA	Alopecia areata	Pre	No	Biological
**18**	F	> 12 y	Systemic JIA	Alopecia areata	Pre	No	Biological
**19**	M	> 12 y	ERA HLA-B27+	Vitiligo + AIT	Pre	No	Biological
**20**	F	6–12 y	Systemic JIA	Vitiligo + AIT	Pre	No	Biological
**21**	F	< 6 y	eoJIA-ANA+	Vitiligo + AIT	Pre	No	Biological

AIT, autoimmune thyroiditis; COVID, documented SARS-CoV-2 infection (positive RT-PCR or anti–SARS-CoV-2 IgG/IgM serology recorded in the medical record); csDMARDs, conventional synthetic disease-modifying antirheumatic drugs; eoJIA-ANA+, ANA-positive extended oligoarticular JIA; ERA, enthesitis-related arthritis; F, female; IBD, inflammatory bowel disease; M, male; Morphea, localised scleroderma; pJIA, polyarticular JIA; RF, rheumatoid factor. The absence of documented SARS-CoV-2 infection does not exclude infection, since serology was not systematically performed across the cohort.
